# Identification of protein-RNA interaction sites using the information of spatial adjacent residues

**DOI:** 10.1186/1477-5956-9-S1-S16

**Published:** 2011-10-14

**Authors:** Wei Chen, Shao-Wu Zhang, Yong-Mei Cheng, Quan Pan

**Affiliations:** 1 College of Automation, Northwestern Polytechnical University, 710072, Xi'an, China

## Abstract

**Background:**

Protein-RNA interactions play an important role in numbers of fundamental cellular processes such as RNA splicing, transport and translation, protein synthesis and certain RNA-mediated enzymatic processes. The more knowledge of Protein-RNA recognition can not only help to understand the regulatory mechanism, the site-directed mutagenesis and regulation of RNA–protein complexes in biological systems, but also have a vitally effecting for rational drug design.

**Results:**

Based on the information of spatial adjacent residues, novel feature extraction methods were proposed to predict protein-RNA interaction sites with SVM-KNN classifier. The total accuracies of spatial adjacent residue profile feature and spatial adjacent residues weighted accessibility solvent area feature are 78%, 67.07% respectively in 5-fold cross-validation test, which are 1.4%, 3.79% higher than that of sequence neighbour residue profile feature and sequence neighbour residue accessibility solvent area feature.

**Conclusions:**

The results indicate that the performance of feature extraction method using the spatial adjacent information is superior to the sequence neighbour information approach. The performance of SVM-KNN classifier is little better than that of SVM. The feature extraction method of spatial adjacent information with SVM-KNN is very effective for identifying protein-RNA interaction sites and may at least play a complimentary role to the existing methods.

## Background

The interaction between Protein and RNA play an essential role in many cellular processes, such as regulation of gene expression, protein synthesis, as well as replication and assembly of many viruses [1,2]. Although there are some literatures to investigate the mechanisms by which protein bind to DNA, the identification of RNA-binding proteins, especially their binding sites in residue level is quite poor. The mechanism that RNAs interact with protein and their binding sites is still a major challenge in the Post-genome era. The ability to identify the specific amino acid that contribute to the specificity of protein-RNA interaction can broaden our understanding of the molecular recognition, the mechanisms of many important biological processes and guide for the mutant design and drug design [3-5]. Unfortunately, the experimental methods such as NMR, immunoprecipitation, and crystallography are all both expensive and laborious for determining the protein-RNA interaction sites. Therefore, there is necessary to develop potential computational methods for predicting the protein-RNA interaction sites.

Recently, the number of the structures of known protein-RNA complexes solved by X-ray crystallography and other high throughput technical is increasing, which supply more potential data resources for developing computing methods. Although some computing methods have been triggered to predict protein-RNA interaction sites for complementing experimental data, these methods are mainly based on sequence and structure information. With single sequence and secondary structure information, Jeong [6] trained an artificial neural network classifier to identify Protein-RNA interacting residues. Terribilini [7] developed a Naive Bayes-based method to predict protein-RNA interaction sites with single protein sequence information. Different from Jeong and Terribilini, Wang and Brown [8] developed a SVM-based tool named BindN for prediction of DNA and RNA binding sites based on the information of side chain pKa value, hydrophobicity index and molecular mass of an amino acid. Later, Wang [9] also develop a SVM-based method PRINTR to identify RNA binding sites in proteins using different feature information, such as single sequence, multiple sequence alignment, secondary structure and solvent accessibility. Manish [10] developed an improved method with evolutionary information and SVM to predict protein-RNA interaction sites. Though these methods have made some progress, developing effective computing methods for predicting protein-RNA interaction sites is also a hotspot area.

Inspiring by the work of Wang [11] who used spatial sequence profile to predict protein-protein interaction sites, we proposed a novel feature extraction method which integrates spatial adjacent residues information and protein structure information, and introduced SVM [12] and Nearest Neighbour classifier [13] algorithm to predict protein-RNA interaction sites. It well known that in the cell, RNA binding protein are showed in a three-dimensional structure or in the form of polymer, thus, in this paper, we consider the influences of the spatial adjacent residues of the target residue.

## Methods

### Dataset

In order to evaluate the performance of the predictor capturing the properties of residues located on a protein-RNA interface, a dataset PRNA79 was established with the dataset RNA109 used by Terribilini [7]. First, we retrieved 59 RNA-binding protein complexes (RBP) solved by X-ray crystallography with a better resolution than 3.5 Å in the PDB. Then, the protein chains with sequence identity value >30% were removed. Last, a protein residue and a RNA base are considered in contact if the closest distance between any pair of heavy atoms from them is less than 5 Å and the residue in protein is defined as interface residues. According above definition, we yielded the dataset PRNA79, which contain 79 non-redundant protein RBP chains and 6157 interface residues.

### SVM-KNN algorithm

In the present work, SVM-KNN algorithm was used to construct predictors to determine whether a residue is an interaction site or not. SVM-KNN was an improved method combination the advantage of SVM and k-Nearest Neighbour (KNN) [14], and it has been successfully applied to many pattern recognition problems. Because the SVM classifier can be regarded as a 1-NN classifier in which only one representative point is selected for each class, and the classification performance of the samples near the optimal classification hyperplane is not very perfect. After integrating K-NN algorithm, the SVM-KNN algorithm chooses more representative points rather than one for the samples near the optimal classification hyper-plane, and it can reduce the classification error caused by just selecting one representative point respectively.

To solve the prediction problem, the SVM-KNN adopts the same training process as SVM to obtain the support vectors and parameters respectively, which were used to construct the decision function:(1)

Where *b* is a bias, *K*(*x*, *x_i_*) denotes the kernel function and *a_i_* is coefficient, the kernel function is RBF in this paper. The difference between SVM and SVM-KNN is at the class phase. The SVM-KNN should compute the distance from the test sample to the optimal hyperplane of SVM in feature space firstly, then according the distance to make a decision, if the distance is greater than the given threshold, the test sample would be classified by SVM; otherwise, the class of the sample will be up to the KNN algorithm, which can be expressed as follows:(2)

Where *C*_1_ and *C*_2_ are weight parameters used to balance the sample number difference in order to improve the performance, *T* is threshold value.

### Feature representation

If a protein sequence has N residues, its PSSM (Position-Specific Iterated Matrix) is a 20 × N matrix which can be generated by PSI-BLAST [15] programme. Here, the default values of PSI-BLAST were used to search the Swissprot database [16] which contains 348,901 protein sequences, and the substitution matrix is BLOSUM62 [17]. Meanwhile, the secondary structural unit information and accessibility solvent area (ASA) of each residue in each protein chain were calculated by DSSP [18].

### Spatial adjacent residues profiles

Considering that the RNA-binding proteins exist in the form of three-dimensional conformation or polymer and the influence of adjacent residues, we use a sliding window of size *w* to represent the target residue based on spatial adjacent residues profiles for the *i*-th target residue in the protein sequence, then the *i*-th target residue can be represented by the following 20*w* dimension vector:(3)

where *pssm_i_* is the sequence profile of i-th target residue, *pssm*_*i*, *j*_ is the sequence profile of j-th nearest spatial adjacent residue of i-th residue, and the adjacent residues are sort in ascending order according the distance of the target residue with other residues which were calculated based on their three-dimensional structure. For the PRNA79 dataset, selected *w*=15, thus, each target residue is represented by a 300D vector. Conveniently, the feature set based on the spatial adjacent residues profiles can be wrote written as *SpaPF.*

### Spatial adjacent residues weighted accessibility solvent area

In order to consider and measure the variety and influences of accessibility solvent area when the protein and RNA interact with each other, the feature extraction approach of spatial adjacent residue weighted accessibility solvent area was introduced. The i-th amino acid target residue in protein sequence can be described by the accessibility solvent area of target residue and spatial adjacent residues within a sliding window of size w, written as:(4)

where *ASA_i_* is the accessibility solvent area of *i*-th target residue, and *ASA_i_*_,_*_j_* is the solvent accessibility of *j*-th nearest spatially adjacent residue of *i*-th residue. Selected *w*=15, each target residue is represented by a 15D vector. Conveniently, the feature set based on the spatial adjacent residues weighted accessibility solvent area information can be wrote written as *SpawASA.*

### Spatial adjacent residues secondary structure information

According to definition of DSSP, the secondary structural units are classified to alpha helices, beta strands and coils. Then the *i*-th target residue can be represented the following feature vector, which integrates the secondary structure information of target residue and spatial adjacent residues within a window *w:*(5)

where *f*_*i*, *H*_, *f*_*i*, *E*_, *f*_*i*, *C*_ are the occurrence frequencies of helix content, beta strand content and coil content within the window *w* respectively. Conveniently, the feature set based on the spatial adjacent residues secondary structure information can be wrote written as *SpaSecond.*

### Evaluation of prediction system

Generally speaking, the jackknife test is widely used to examine the prediction performance of the classifier. The cross-validation by jackknife is thought the most objective and rigorous way in comparison with *q-*fold cross test or independent data set test [19], however, it have a big computational power, especially for a large dataset. In this paper, we used 5-fold cross-validation (5CV) test approach. The total prediction accuracy (*Q*), the sensitivity (*Sen*), the specificity (*Spe*) and Matthew’s Correlation Coefficient (*MCC*) [20] were used to evaluate prediction system.(6)(7)(8)(9)

Here, N is the total number of residues, TP is the number of RNA-binding residues predicted correctly; FP is the number of RNA-binding residues predicted wrongly; TN denotes the number of non-RNA-binding residues predicted correctly; FN denotes the number of non-RNA-binding residues predicted wrongly.

## Results and discussion

The optimal parameters for SVM-KNN are very important which have a vital influence on the performance of the classifier. In this work, the kernel function is RBF, the parameters C and *γ* were chosen in 5CV test, the weighted parameters *C*_1_ and *C*_2_ were set based on the number of interaction residues (*N*_+_) and the number of noninteraction residues (*N*_-_ ) in the training set. For the dataset PRNA79, the optimal parameters are *C* = 1, *γ* = 0.0625, *C*_1_ = 1 and *C*_2_ = *N_-_/N*_+_ = 1.5 .

The three feature sets of *SpaPF*, *SpaASA* and *SpaSecond* feature were employed to train SVM-KNN classifier and SVM classifier respectively. The classification results in 5CV test are summarized in Table 1. From Table 1, we can find that the performance of *SpaPF* is better than that of other two feature sets. The overall accuracy of *SpaPF* with SVM-KNN is 78.00%, which is 14.7%, 10.0% higher than that of *SpaSecond* and *SpaASA* respectively. The MCC of *SpaPF* with SVM-KNN is 0.32, 0.23 higher than that of *SpaSecond* and *SpaASA* respectively. The results show that the *SpaPF* includes more information about the protein-RNA interaction sites due to the profile contained the conservation information through multiple sequence alignment, which also has been demonstrated by other work [9]. Meanwhile, we can also find that the performance of SVM-KNN classifier is little better than that of SVM. It shows that the SVM-KNN can improve classification performance in some degree compared with SVM.

**Table 1:** Results of different feature sets with SVM-KNN and SVM

Classifier	Feature set	*Spe* (%)	*Sen *(%)	*MCC*	Accuracy (%)
SVM-KNN	*SpaSecond*	75.47	44.34	0.21	63.28
	
	*SpawASA*	86.74	38.86	0.30	68.00
	
	*SpaPF*	88.18	62.14	0.53	78.00

SVM	*SpawASA*	92.68	27.27	0.27	67.07
	
	*SpaPF*	87.31	62.37	0.52	77.55

### Selection of window width w

**Figure 1:**
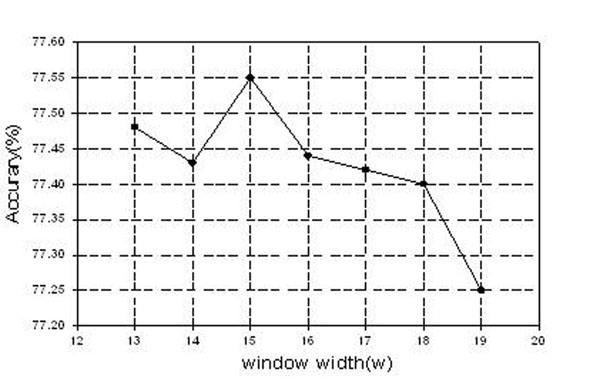
The relationship between the window width *w* (x-axis) and the prediction accuracy (y-axis) with SVM for *SpaPF*.

The window size has some effect to the prediction performance. If window size selected too short, it would lose some important classification information. But if the window size selected too long, it will include more noise information. Unfortunately, there is no rule guiding the window size selection. In this work, in order to find the optimal window size, we test different window length from 13 to 19 with PRNA79 dataset using SVM. The results of different window lengths for *SpaPF* feature in 5-fold cross validation test (5CV) are shown in Figure 1.

From Figure 1, we can see that the prediction performance in 5CV test changes with the window width, and the total accuracy is not always monotonous increasing (or decreasing) with window size. The best result can be arrived when the window width size equals 15.

### Comparison with other feature extraction methods

In order to evaluate our feature extraction method, the performance of our method was also compared with other existing feature extraction methods. The comparison results with SVM-KNN and 5CV test for PRNA79 dataset are summarized in Table 2. From Table 2, we can see that the total accuracy of *SpaPF* and *SpawASA* is 1.4%, 4.72% higher than that of feature extraction methods of sequence neighbour residues profiles (*SeqProfile*) [9] and sequence neighbour residues accessibility solvent area (*SeqASA*) [7]. It means that the *SpaPF* and *SpawASA* contain more protein-RNA interaction site information than *SeqProfile* and *SeqASA*, which will be helpful for predicting the protein-RNA interaction sites. These results show that the novel feature extraction method is quite promising and useful to improve the prediction quality of protein-RNA interaction sites.

**Table 2:** Comparison with other feature extraction methods with SVM

Feature set	Accuracy (%)	MCC
*SeqProfile^a^*	76.60	0.48

*SpaPF*	78.00	0.53

*SeqASA^b^*	63.28	0.16

*SpawASA*	68.00	0.30

a) *SeqProfile*: sequence neighbour residues profiles [9].

b) *SeqASA*: sequence neighbour residues accessibility solvent area [7].

## Conclusions

For distinguishing the interface residues from other surface residues in protein– RNA complexes known structure, a novel feature extraction method integrated spatial adjacent residues information was introduced to predict protein-RNA interaction sites. The results show that feature sets extracted through spatial adjacent residues profiles and accessibility solvent areas contain more information than that of sequence neighbour residues profiles and accessibility solvent areas, the SVM-KNN can improve the performance of predicting protein-RNA interaction sites. The novel feature extraction method integrated spatial adjacent residues information with SVM-KNN is quite promising and may at least play a complimentary role to the existing methods.

## List of abbreviations used

RBP: RNA-binding proteins; NA: Nucleic-acid; PSSM: Position-Specific Iterated Matrix; *SpaPF*: Spatial adjacent residues profiles; *SpawASA*: Spatial adjacent residues weighted accessibility solvent area; *SpaSecond*: Spatial adjacent residues secondary structure information; *SeqProfile*: Sequence neighbour residues profiles; *SeqASA*: sequence neighbour residues accessibility solvent areas.

## Competing interests

The authors declare that they have no competing interests.

## Authors' contributions

WC conceived the study, performed sample preparation and data acquisition. SWZ supplied methodological expertise and rewrite the manuscript. Both authors took part in the design of the study and were critically involved in manuscript drafting. All authors read and approved the final manuscript.
